# Hepatoblastoma in an 11-year-old: Case report and a review of the literature

**DOI:** 10.1097/MD.0000000000005858

**Published:** 2017-01-13

**Authors:** Irina B. Pateva, Rachel A. Egler, Duncan S. Stearns

**Affiliations:** Department of Pediatrics, Case Western Reserve University, Rainbow Babies and Children's Hospital, Cleveland, OH.

**Keywords:** case report, hepatoblastoma, liver tumors

## Abstract

**Rationale:**

Hepatoblastoma is a rare malignancy. Approximately 100 cases are diagnosed yearly in the United States. The highest incidence occurs in infants and in children younger than 5 years. Cases involving patients older than 5 years are very rare. We describe the case of a patient who was diagnosed with hepatoblastoma at an atypical age of presentation for this type of malignancy. We also performed Ovid MEDLINE search for hepatoblastoma and epidemiology reports occurring in children between the ages of 5 and 18 years. In this article we review the epidemiology and summarize case reports published between 1997 and 2012 of patients with hepatoblastoma, who were older than 5 years.

**Patient concerns and diagnosis:**

Our patient is an 11 year old boy with stage IV hepatoblastoma with lung and omental metastases at diagnosis.

**Interventions:**

The patient received 7 cycles of chemotherapy following the treatment plan of COG protocol AHEP 0731, off study. He also had tumor resection and omentectomy and achieved complete remission.

**Outcomes:**

He later had disease recurrence and after undergoing treatment with different modalities, ultimately died of his disease. Review of SEER program data shows that the incidence of hepatoblastoma in children above the age of 5 years is too infrequent to be calculated. Literature review revealed 13 cases of patients diagnosed at age older than 5 years. Most cases were published due to unusual associations and/or complications. There are no obvious unifying characteristics for these cases, although there may be a slight male preponderance and many patients in this selected series presented with elevated Alpha-fetoprotein.

**Lessons:**

The reported case is rare, given the very low incidence of hepatoblastoma outside of infancy. A systematic review of characteristics and outcomes for patients older than 5 years who are enrolled in cooperative group hepatoblastoma trials may reveal important information about the epidemiology and tumor biology in this rare patient population.

## 1 Introduction

Hepatoblastoma (HB) is a rare malignancy, approximately 100 cases are diagnosed yearly in the USA.^[1]^ The most recent SEER data for the period 2002–2008 demonstrates that the highest incidence of hepatoblastoma is in the 0 to 4 years age group, with 10.5 and 5.2 cases per million children <1 and 1 to 4 years. In patients older than 5 years, cases of hepatoblastoma are very rare. The estimated incidence is 0.1 cases per million children in the age group of 5 to 9 years and sometimes reported as “too infrequent to be calculated.”^[1]^

The epidemiology of HB has not been studied extensively. Although its incidence over the last several decades has been well described, as well as certain associations with genetic syndromes and parental environmental exposures, risk factors *per se* have been mainly suggested by case reports. Some of the well-known associations of HB are with Beckwith–Wiedemann, parental exposure to metals and very low birth weight (VLBW). HB has been reported in single instances in association with fetal alcohol syndrome,^[2]^ oral contraceptive use during pregnancy,^[3]^ and maternal liver transplantation with immunosuppressive therapy.^[4]^

Spector et al^[5]^ and Reis et al^[6]^ evaluated HB incidence and trends specifically among children 0 to 4 years in the United States from 1975 through 1999 in the 9 reporting areas of SEER.^[5,6]^ The overall incidence rate of HB in this age group rose from 2.59 per million in 1975–1979 to 5.27 in 1995–1999, which represented a statistically significant 3.9% annual rise in incidence. The rate of HB was slightly higher in males compared to females and in blacks compared to whites. There was a significant annual rise in incidence for males, females, and whites.^[5]^ The average annual percent change (AAPC) for blacks suggested a rise in incidence but was not significant. The incidence of HB is vanishingly small, 0.3 cases per million or less, at ages older than 4 years for the period 1975–1999.^[6]^

Given the noted increase in incidence of HB between 1975 and 1999 and paucity of epidemiologic studies, a cooperative group study—COG AEPI04C1—was designed for patients with HB in the 0 to 4 age group. The aims of the study were to investigate exogenous and endogenous risk factors for HB with special emphasis given to risk factors for the development of HB among children with low birth weight.^[7]^ The study results have not been published yet and we believe that these will reveal new findings and insights into the risk factors and biology of HB in infants and young children. However, the epidemiology and biology of HB in the older patient population still remain to be explored.

In this article, we report a case of an 11-year-old boy diagnosed with stage IV hepatoblastoma with lung metastases and extensive omental studding, which is an infrequent location for metastatic disease. We review briefly the epidemiology of hepatoblastoma and summarize 13 cases of patients between the ages of 5 and 18 years, reported in the English literature over a period of 16 years (1997–2012).

The University Hospitals Cleveland Medical Center Institutional Review Board reviewed this case report and determined that it did not qualify as human subjects research according to Federal regulations; therefore, a formal approval was not necessary. A HIPPA waiver was granted for the individual patient's information to be reviewed.

## 2 Case report

An 11 y/o Hispanic male, who was previously healthy, presented to his primary care physician with persistent abdominal pain of several months. He was found to have a right-sided abdominal mass on physical examination. An abdominal ultrasound was performed and revealed the presence of a large mass, extending inferiorly from the right lobe of the liver and measuring approximately 11.6 × 11.4 × 9.6 cm. An abdominal CT confirmed the presence of a large hepatic mass involving segments 5 and 6 of the liver and peritoneal metastases. Alpha-fetoprotein (AFP) was elevated; it was 35,084 ng/mL (reference range 0–15 ng/mL). Biopsy of omental metastases revealed epithelial hepatoblastoma, embryonal type.

Treatment was started with 1 cycle of vincristine/irinotecan (VI) followed by 4 cycles of cisplatin and 5 fluorouracil, vincristine, doxorubicin (C5VD). Our patient's tumor was classified as PRE-TEXT II at diagnosis and was POST-TEXT I after 5 cycles of chemotherapy. The patient had surgical resection after the initial 5 cycles of chemotherapy. His AFP decreased from 35,084 ng/mL at diagnosis to 2612 ng/mL after 2 cycles of chemotherapy and to 162 ng/mL prior to surgery. The AFP came down to 28 ng/mL post-surgery and it normalized completely 1 month later. He received 2 more cycles of C5VD postoperatively. He did not require surgical resection for his lung lesions; they completely resolved with chemotherapy. The patient was followed with CT scans of chest, abdomen, and pelvis for disease recurrence after the end of therapy. His AFP started increasing approximately 2 months post end of therapy (initially was 26 μg/L). At first, there was no radiographic evidence of disease despite a 4-month upward trend of his AFP. At that time, a very small lung lesion was found on CT of chest. Three months later, a repeat chest CT and an MRI of his abdomen detected enlarging lung lesions and a hepatic lesion. At that time, his AFP had increased to 145 ng/mL. Biopsy of liver lesion proved recurrent hepatoblastoma.

Treatment postrelapse was initiated with vincristine, irinotecan, and temozolomide (VIT) for 2 cycles and the patient had stable disease. After 2 more cycles, disease progression was noted. In light of these findings, 2 cycles of ifosfamide, carboplatin, and etoposide (ICE) were also given; however, the patient had disease progression with an increase in size of a hepatic lesion and new subdiaphragmatic lesions found on MRI. Of note, for our patient, the MRI was found to be a more useful imaging modality for identifying the liver and subdiaphragmatic lesions at relapse and following their progression. He underwent radiofrequency ablation (RFA) of the liver lesion and the diaphragmatic tumor implants. After the RFA, his AFP normalized and remained normal for 3 months. He continued with chemotherapy—irinotecan, temozolomide, and temsirolimus. Approximately 9 months after the RFA, an increase in size of the subdiaphragmatic, liver, and lung lesions was detected, together with rising AFP. The patient underwent transarterial chemoembolization (TACE) with doxorubicin for his liver lesions and had a significant decrease in the AFP post-procedure. Because he still had lung lesions, systemic chemotherapy continued. He was given a PARP inhibitor and temozolomide on a phase 1 clinical trial followed by axitinib. His disease progressed with increase in size of the hepatic lesion and IVC involvement with intracardiac/right atrial extension. He received palliative radiation to the areas of disease involvement of his liver, IVC, and right atrium. Subsequently, he received hospice care and died of his tumor 44 months from his initial diagnosis.

## 3 Discussion

Here, we review the epidemiology of hepatoblastoma (HB), the most common associations, and summarize case reports for patients with HB above the age of 5 years.

Fifteen percent of all abdominal tumors in childhood are primary liver tumors, 66% of these are malignant, the most common being hepatoblastoma (HB).^[8]^

About 100 cases of hepatoblastoma are diagnosed annually in the USA.^[1]^

Review of SEER database for the 2002–2008 period demonstrates that among patients younger than 5 years, hepatoblastoma accounts for 91% of primary hepatic malignancy cases. Age-specific incidence was reported to be highest in the 0- to 4-year-old age group. The occurrence of hepatoblastoma is too infrequent in children older than 5 years and the actual incidence could not be calculated.^[1]^ In comparison, in the 15 to 19 years age group, hepatocellular carcinoma accounts for 87% of cases.

International data from different regions in the world report no significant variations in the incidence of HB between different nations.^[1,9,10]^

Hepatoblastoma is slightly more frequent in males and its incidence is higher in patients with several genetic and cancer predisposition syndromes: Beckwith–Wiedeman, Familial Adenomatous Polyposis, and Trisomy 18.

Another factor which is strongly linked with higher incidence of HB is very low birth weight (generally considered when the newborn is < 1500 g).

Pre-eclampsia, poly- or oligohydramnios, high maternal prepregnancy weight, and treatment for infertility in women were also reported to correlate with higher hepatoblastoma occurrence in their children. Other associations have been observed between tobacco smoking of both parents pre- and postconception and HB and parental occupational exposure to metals.^[1,11]^

Several clinico-pathological factors relevant to long-term outcome in newly diagnosed patients with HB have been identified. Maibach et al^[12]^ reported that among those factors, correlating significantly with reduced event-free survival is low or very high AFP at diagnosis—when it is lower than 100 ng/mL or if it is higher than 1.2 × 10^6^ ng/mL. Elevated AFP is a known hallmark of active disease in patients with HB. It is generally significantly elevated with large tumor burden and its level decreases with therapy. It is frequently utilized as a marker of disease activity and response to therapy. Patients who are found to have a quick drop in AFP and normalization early in their therapeutic course tend to have better outcomes.

Metastatic disease at presentation, PRETEXT IV, age > 5 years and some other clinical and histopathological characteristics were also found to correlate with poor outcomes.^[12]^ De Ioris et al^[13]^ also identified patients with HB and low serum AFP (< 100 ng/mL) as high-risk subgroup with extensive disease at diagnosis, poor response to therapy, and poor survival.

In contrast to previously described clinico-pathological indicators known to correlate with the poor outcomes, molecular profiling of HB had not been performed until recently. Identification of biomarkers with a goal to improve risk stratification, recognize prognostic implications, and potentially influence treatment decisions has been sought of late. Sumazin et al analyzed 88 HBs and revealed 3 risk-stratifying molecular subtypes that are characterized by differential activations of hepatic progenitor cell markers and metabolic pathways. For example, high-risk tumors were characterized by up-regulation of certain genes and proteins, one of which was high AFP expression and high coordinated expression of oncofetal proteins and stem cell markers. Low-risk tumors had low level of expression and activity of these genes and proteins. Further analyses of targeting these genes in a prospective study of 35 HBs suggested that these candidate biomarkers have the potential to improve risk stratification and guide treatment decisions for HB patients at diagnosis.^[14]^

Our patient did not have any of the above-mentioned clinico-pathological risk factors associated with higher incidence of HB. Upon review of his past medical history, we found out that he was born prematurely at 34 weeks. Nevertheless, with no history of VLBW, he had a brief and uncomplicated NICU course. He did not have other symptoms, signs or physical exam findings to suggest a genetic syndrome or a cancer predisposition condition. In terms of risk factors associated with poor outcomes, his AFP level at diagnosis did not place him in the unfavorable category; however, he had metastatic disease and was older than 5 years. His case is rare because of his age at diagnosis and extensive omental metastases, not commonly found as a site of disease in patients with HB.

Here, we summarize 13 cases of patients with HB older than 5 years (Table 1). The cases were from different countries and were reported over a period of 16 years (1997–2012) due to different associations or complications during treatment.

**Table 1:**
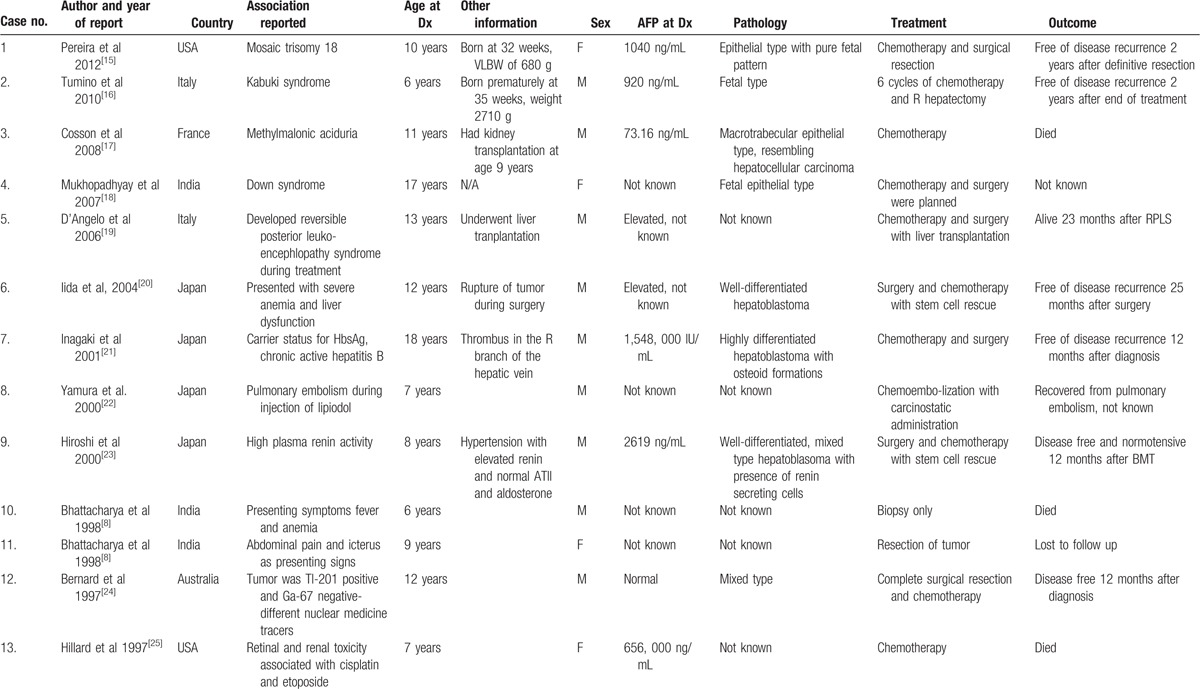
Case reports of patients with hepatoblastoma.

There were no unifying characteristics of these patients, other than male preponderance and elevated AFP at diagnosis. It is worth noting that they were reported from all over the world, with no particular geographic predilection of certain pathological subtypes or comorbidities. Three of the cases were reported as associations with genetic syndromes, not previously described: Down syndrome, Kabuki, and partial trisomy 18. One of the patients who presented at the same age as our patient had an underlying rare metabolic disorder—metylmalonic aciduria. Two of the patients were born prematurely, although only 1 had a VLBW.

Different pathological subtypes of the tumors were noted. One of the most common histological types of tumors was epithelial type, which is the same as in our patient. Of note, in 1 of those 3 tumors, cells which resembled hepatocellular carcinoma were seen. To our knowledge, our patient did not have cells that are typical of, or resemble, hepatocellular carcinoma. However, based on the biological behavior of his tumor, with short-lived response to first-line therapy for HB, there was a question of whether he had a transitional cell liver tumor, which is a relatively new entity that has been described in the recent years. This speculation was based on the aggressiveness of his disease and was not proven by histological or other analyses. At least 3 of the cases had well-differentiated tumor types and, interestingly, in one of the tumors renin secreting cells were found. One of the well-differentiated tumors had osteoid formations. There a few cases that did not have pathology of tumors reported; thus, we cannot make conclusions about overall correlation of pathological subtypes and patient outcomes.

The case reports have described the different therapeutic modalities which were used. Most of the patients had surgical resection, 1 patient had liver transplant, and several patients received different chemotherapy agents. Two patients had high-dose chemotherapy with stem cell rescue. One patient had chemoembolization. Going back to our patient, his treatment consisted of a multitude of different agents and modalities. He received several different chemotherapeutic regimens, had surgery, radiofrequency ablation, trans-arterial chemoembolization, and palliative radiation. From the reported cases, we cannot make a conclusion about certain types of treatments and outcomes. This is due to this small case series and also the focus when reporting these patients had not been to review in detail the treatments utilized, but rather complications that occurred or new associations.

Outcomes in these case series were variable with no clear evidence of favorable or unfavorable characteristics.

## 4 Conclusion

In summary, the reported case is rare given the very low incidence of hepatoblastoma outside of infancy. Our literature search revealed 13 other cases of patients with HB above the age of 5 years published over a period of 16 years. These cases were reported due to complications of treatment or associations not being described previously. We suspect that there are other cases of older patients with HB, who were participants of cooperative group clinical trials, and have not been reported separately.

Although epidemiologic studies have been initiated to investigate exogenous and endogenous risk factors for HB in infants and young children in the age 0 to 4 years, the influence of older age at diagnosis on disease course and survival of patients with HB has not been well studied. Our case highlights the importance of considering a systematic approach to study characteristics and outcomes of patients older than 5 years enrolled in cooperative group hepatoblastoma trials. This will provide insights into tumor biology in this rare population and rationale for future risk stratification schemas and new risk-adapted therapeutic strategies.

## References

[1] Spector LG Birch J The epidemiology of hepatobalstoma. Pediatr Blood Cancer 2012;59:776–9. 22692949 10.1002/pbc.24215

[2] Khan A Bader JL Hoy GR Hepatoblastoma in child with fetal alcohol syndrome. Lancet 1979;1:1403–4. 10.1016/s0140-6736(79)92035-x 87858

[3] Otten J Smets R De Jager R Hepatoblastoma in an infant after contraceptive intake during pregnancey. N Engl J Med 1977;297:222. 10.1056/nejm197707282970417 195205

[4] Roll C Luboldt HJ Winter A Hepatoblastoma in a 2-year-old child of a liver-transplanted mother. Lancet 1997;349:103 1997. 10.1016/S0140-6736(05)60887-2 8996429

[5] Spector LG Feusner JH Ross JA Hepatoblastoma and low birth weight. Pediatr Blood Cancer 2004;43:706. 15390302 10.1002/pbc.20122

[6] Ries LA Eisner MP SEER Cancer Statistics Review, 1973–1999. Bethesda, MD:National Cancer Institute; 2002.

[7] Spector LG AEPI04C1: Low Birth Weight & Other Risk Factors for Hepatoblastoma a Groupwide Protocol, pp 4–9.

[8] Bhattacharya S Lobo FD Pai PK Hepatic neoplasms in childhood—a clinicopathologic study. Pediatr Surg Int 1998;14:51–4. 9880696 10.1007/s003830050434

[9] de Fine Licht S Schmidt LS Rod NH Incidence of HB in the Nordic countries. Int J Cancer 2012;131:E555–61. 22095187 10.1002/ijc.27351

[10] Lee CL Ko YC Survival and distribution pattern of childhood liver cancer in Taiwan. Eur J Cancer 1998;34:2064–7. 10070312 10.1016/s0959-8049(98)00281-0

[11] Litten JB Tomlinson GE Liver tumors in children. Oncologist 2008;13:812–20. 18644850 10.1634/theoncologist.2008-0011

[12] Maibach R Roebuck D Brugieres L Prognostic stratification for children with hepatoblastoma: the SIOPEL experience. Eur J Cancer 2012;48:1543–9. 22244829 10.1016/j.ejca.2011.12.011

[13] De Ioris M Brugieres L Zimmermann A Hepatoblastoma with a low serum alpha-fetoprotein level at diagnosis: the SIOPEL group experience. Eur J Cancer 2008;44:545–50. 18166449 10.1016/j.ejca.2007.11.022

[14] Sumazin P Chen Y Treviño LR Genomic analysis of hepatoblastoma identifies distinct molecular and prognostic subgroups. Hepatology 2017;65:104–21. 27775819 10.1002/hep.28888

[15] Pereira EM Marion R Ramesh KH Hepatoblastoma in a mosaic trisomy patient. J Pediatr Hematol Oncol 2012;34:e145–8. 22469941 10.1097/MPH.0b013e3182459ee8

[16] Tumino M Licciardello M Sorge G Kabuki syndrome and cancer in two patients. Am J Med Genet A 2010;152A:1536–9. 20503331 10.1002/ajmg.a.33405

[17] Cosson MA Touati MG Lacaille F Liver hepatoblastoma and multiple OXPHOS deficiency in the follow up of a patient with methylmalonic aciduria. Mol Genet Metab 2008;95:107–9. 18676166 10.1016/j.ymgme.2008.06.007

[18] Mukhopadhay P Kundu SS Banerjee A Adult HB in a female down's. JAPI 2007;55:242–3. 17598340

[19] D’Angelo P Farruggia P Lo Bello A Reversible posterior leukoencephalopathy syndrome, report in 2 simultaneous cases in children. J Pediatr Hematol Oncol 2006;28:177–81. 16679945 10.1097/01.mph.0000210406.82050.07

[20] Iida T Suenaga M Tacheuchi Y Successful resection of a ruptured HB prior to chemotherapy: report of a case. Surg Today 2004;34:710–4. 15290405 10.1007/s00595-004-2766-9

[21] Inagaki M Yagi T Urushihara N Successfully resected hepatoblastoma in a young adult with chronic hepatitis B: report of a case. Eur J Gastroenterol Hepatol 2001;13:981–4. 11507368 10.1097/00042737-200108000-00020

[22] Yamaura K Higashi M Akioshi K Pulmonay lipiodol embolism during transcatheter arterial chemoembolization for hepatoblastoma under general anesthesia. Eur J Anesthesiol 2000;17:704–8. 10.1046/j.1365-2346.2000.00759.x 11029570

[23] Hiroshi M Akinobu T Sachiyo K Renin-producing hepatoblastoma. J Pediatr Hematol Oncol 2000;22:78–80. 10695827 10.1097/00043426-200001000-00015

[24] Bernard E Wayne N Howman-Giles R Tl-201 positive, Ga-67 negative hepatoblastoma: a case report of a 12 year old boy. Clin Nucl Med 1997;22:855–7. 9408645 10.1097/00003072-199712000-00006

[25] Hillard L Berkow R Watterson J Retinal toxicity associated with cisplatin and etoposide in pediatric patients. Med Pediatr Oncol 1997;28:310–3. 9078334 10.1002/(sici)1096-911x(199704)28:4<310::aid-mpo12>3.0.co;2-g

